# Isolated cervical extraosseous intradural chordoma attached to the C5 nerve root: a case report

**DOI:** 10.1186/s41016-019-0170-y

**Published:** 2019-09-09

**Authors:** Juliana Rotter, Kyle Mueller, Ashley MacConnell, Jason McGowan, Steven Spitz

**Affiliations:** 1 Georgetown University School of Medicine, 3700 Reservoir Rd, Washington, DC 20007 USA; 2 Department of Neurosurgery, Medstar Georgetown University Hospital, 3800 Reservoir Rd, Washington, DC 20007 USA; 3 Department of Neurosurgery, University of Pittsburgh Medical Center, 200 Lothrop St. Ste B400, Pittsburg, PA 15213 USA

**Keywords:** Chordoma, Extraosseous, Nerve root, Cervical spine, Intradural extramedullary

## Abstract

**Background:**

As chordomas are slow growing and locally invasive with high recurrence rates, initial recommendations include complete surgical resection with or without radiation therapy. A large proportion of recurrences occur years after initial resection necessitating lengthy follow-up. The novel biomarker brachyury and the repurposing of pharmaceutical products have the potential to substantially impact long-term recurrence rates.

**Case presentation:**

A 43-year-old woman presented with an isolated, cervical extraosseous intradural extramedullary chordoma attached to a nerve root underwent a C3-5 laminectomy, C3-5 lateral mass screw instrumentation, and mass resection. All symptoms resolved by the 12-month postoperative follow-up visit.

**Conclusions:**

This is the first report of an isolated, cervical extraosseous intradural extramedullary chordoma attached to a nerve root, and this case adds to the previous six Type IV chordomas in the literature. Unfortunately, the very rare form of extraosseous intradural chordoma is poorly understood: the lack of detailed knowledge in how they are differentiated from other forms of chordoma confounds the development of optimal treatment strategies and follow-up guidelines.

## Background

Chordomas are rare, locally malignant neoplasms with high recurrence. Novel biomarkers enable definitive diagnosis, and pharmacotherapies are being studied in advanced disease. The paucity of research of a rare type—extraosseous intradural—prevents complete application of breakthroughs to these patients.

## Case presentation

### Patient presentation

A 43-year-old African American woman with no significant history presented with 2 years of worsening, bilateral arm numbness whose intensity waxed and waned daily. She developed bilateral foot numbness without gait difficulties, weakness, or trauma. Her examination was notable only for bilateral 3+ biceps, brachioradialis, patellar and Achilles reflexes, and Hoffmann signs.

**Fig. 1:**
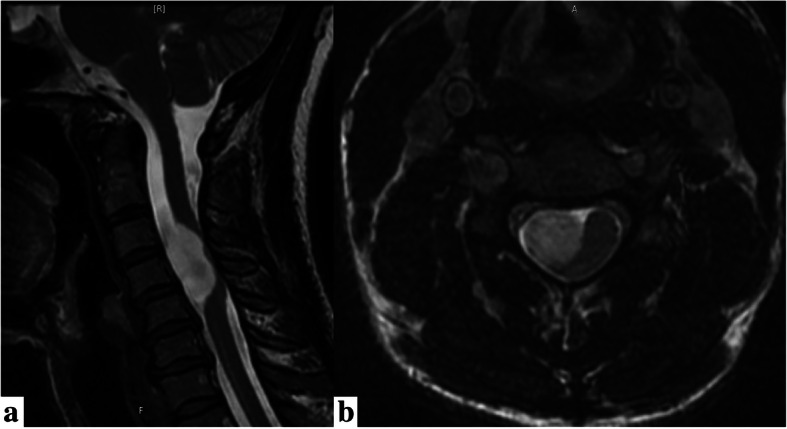
**a** Sagittal and **b** axial preoperative T2 MRI

Cervical spine MRI (Fig. 1) demonstrated a C3-5 right paracentral extramedullary homogenous 2.7 × 1.1 × 1.6 cm lesion that displaced the spinal cord left of midline. A central nidus had intense enhancement without hemorrhage or edema. Additional imaging revealed no other lesions.

### Operation

**Fig. 2:**
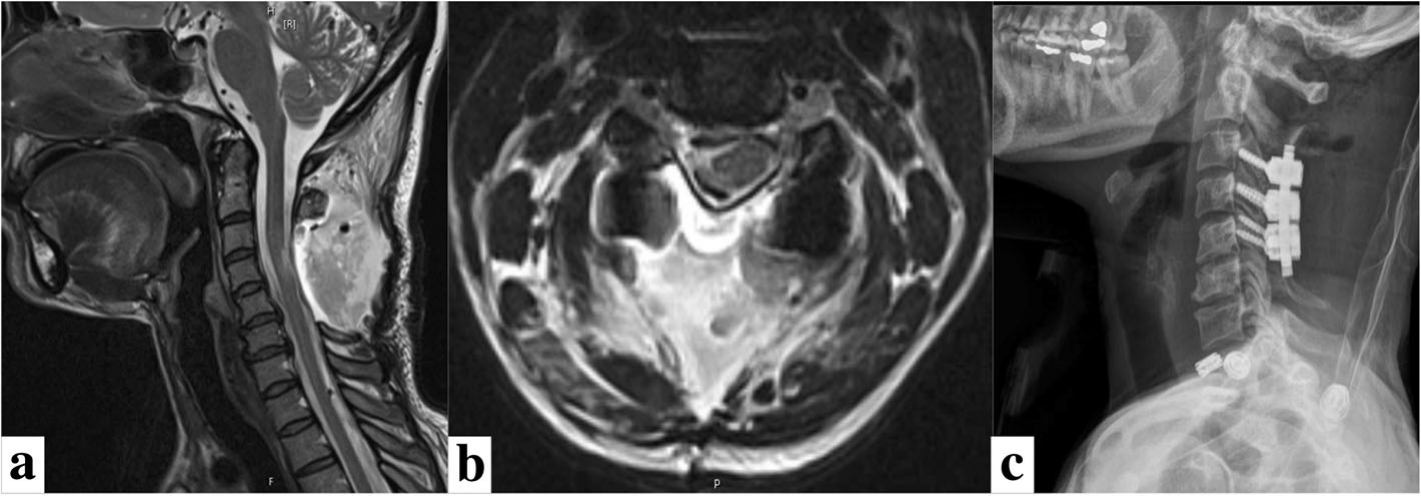
**a** Sagittal and **b** axial postoperative T2 MRI. **c** Lateral X-ray showing gross total resection and stable instrumentation

The patient was positioned prone with the head slightly flexed. A midline incision with subperiosteal dissection then a C3-5 laminectomy with C3-5 lateral mass screw instrumentation was performed. The tumor adhered to the right C5 nerve root. The tumor was completely resected (Fig. 2).

### Histopathology

**Fig. 3:**
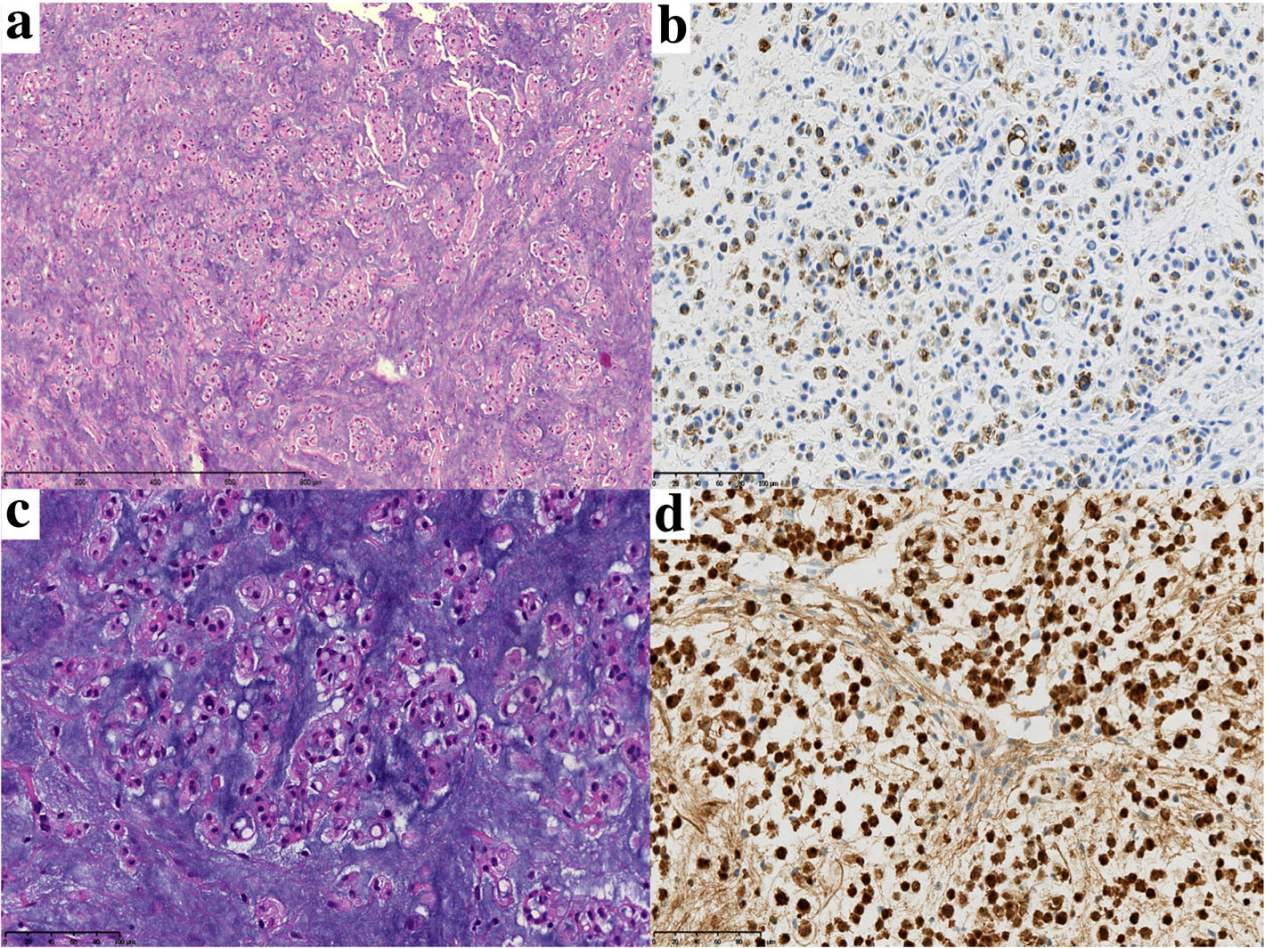
**a** Cell cords and lobules, fibrous septa, and myxoid stroma. **b** Pan keratin stain. **c** Physaliferous cells and myxoid stroma. **d** S100 stain

The tumor stained positively for S100, pan cytokeratin, and vimentin, but negatively for CD10. Multiple institutions deemed routine stains definitive for chordoma, so brachyury stains were omitted (Fig. 3).

### Postoperative course

Her hospital course was uneventful. Radiation oncologists choose to not radiate as there was no specific target. By 10 months, all symptoms resolved except for a small area of minor numbness in the right upper extremity. Though annual MRI surveillance was recommended, she was lost to follow-up.

## Discussion and conclusions

Chordomas are slow-growing, locally invasive tumors arising at the cranial (32%), spinal (32%), and sacral (29%) sites occurring in 8.4 per 10 million patients [1]. Historically hypothesized as originating from notochordal remnants, recent research revealed that some developed from benign notochordal cell tumors (BCNT) [2]. The classification system is based on location: osseous extradural (Type I), extraosseous extradural (Type II), osseous intradural (Type III), and extraosseous intradural (Type IV) chordomas [3].

Signs and symptoms reflect the lesion’s location and include pain in the neck, shoulders, and upper extremities with weakness and occasional myelopathy [4]. Although imaging analysis revealed few unique features, cervical chordoma should be considered in a hypo- or isodense lytic lesion of the vertebral body on CT with a large hyperintense soft-tissue mass on T2-weighted MRI [4]. Chordomas are usually hypo- or isointense on T1-weighted MRI and all enhanced with IV gadolinium contrast [4].

Local aggressiveness necessitates surgery to minimize destruction of bone and surrounding structures. Excision is recommended except in patients with a history of radiotherapy where palliative resection is more appropriate based on a higher complication rate [5]. Recurrence occurs in > 50% patients with a high proportion occurring 5–10 years after resection, so a multi-disciplinary group developed long-term management recommendations [6].

Though biomarkers including S100 and cytokeratin are diagnostically useful, brachyury is specific to notochord-derived tumors. These soft and tan tumors hemorrhage frequently and are highly cellular with a physaliophorous appearance [7]. Additional research may reveal histologic, immunohistochemical, and genomic differences between the four types and between de novo, recurrent, BCNT-derived and metastatic chordomas.

Since designing drugs is predicated upon identifying a specific target, some have proposed developing a therapy for brachyury while others have focused on repurposing existing medications. A study of 2800 drugs showed that digoxin, digitoxin, and bortezomib inhibited growth in chordoma cell lines and primary culture, and many other medications exhibited varying potency [8]. Afatinib, an epidermal growth factor receptor inhibitor used in non-small cell lung carcinoma, was found to degrade brachyury and is being applied to advanced chordoma [9]. Imatinib has effectively treated locally advanced and metastatic platelet-derived growth factor subunit B-positive chordoma [10].

While biomarker and pharmacotherapy discoveries may impact chordoma patients, Type IV has only been reported in six patients so it remains very poorly understood [11]. Two presented with multiple lesions, one spanned multiple regions, and the remainder were confined to one region. Patients underwent total, partial, or piecemeal resection with or without radiation. Follow-up varied.

Chordomas exhibit high recurrence rates even years after resection. Biomarkers and repurposed pharmacotherapies have shown promise in diagnosis and treatment. The rarity of Type IV chordomas prevents understanding of their unique characteristics as distinguished from other types—information required for prognostication and guidelines. Further studies may reveal differences between chordoma variations that would enable appropriate development of personalized treatment plans.

## Data Availability

Not applicable.

## Abbreviations

BCNT: Benign notochordal cell tumors
MRI: Magnetic resonance imaging
